# Spatiotemporal Moiré lattice light fields

**DOI:** 10.1515/nanoph-2024-0562

**Published:** 2025-01-06

**Authors:** An-Zhuo Yu, Wang Zhang, Wei Chen, Yuan Liu, Chao-Qun Ma, Jia-Chen Yang, Yan-Qing Lu

**Affiliations:** National Laboratory of Solid State Microstructures, Key Laboratory of Intelligent Optical Sensing and Manipulation, Collaborative Innovation Center of Advanced Microstructures, College of Engineering and Applied Sciences, Nanjing University, Nanjing 210023, China

**Keywords:** space-time modulation, Moiré effect, diffraction-free

## Abstract

Joint space-time modulation of light fields has recently garnered intense attention for enabling precise control over both spatial and temporal characteristics of light, leading to the creation of space-time beams with unique properties, such as diffraction-free propagation and transverse orbital angular momentum. Here, we theoretically propose and experimentally demonstrate spatiotemporal Moiré lattice light fields by controlling the discrete rotational symmetry of a pulse’s spatiotemporal spectrum. Using a 4*f* pulse shaper and an *x* − *ω* modulation strategy, we generate tunable spatiotemporal Moiré patterns with varying sublattice sizes and confirm their diffraction-free behavior in time-averaged intensities. Additionally, we demonstrate spatiotemporal Moiré lattices carrying transverse orbital angular momentum. These findings provide a novel platform for studying spatiotemporal light–matter interactions and may open new possibilities for applications in other wave-based systems, such as acoustics and electron waves.

## 1 Introduction

Light field manipulation – the exact regulation of light’s properties – has become a fundamental aspect of modern optics. Traditionally, the spatial and temporal modulation of light fields have been treated as relatively independent processes: spatial control addressed parameters such as propagation direction, phase, and amplitude, while temporal control focused on pulse duration and frequency (or wavelength) [1], [2]. Nevertheless, recent advances have highlighted the advantages of simultaneously modulating light in both space and time, rooted in the inherent duality of these dimensions in Maxwell’s equations [3]. This joint space-time modulation has naturally led to the extension of spatially structured light into the space-time domain, resulting in space-time beams with unconventional and unique physical properties. Notable examples include spatiotemporal optical vortices carrying transverse orbital angular momentum [4], [5], [6], [7], [8], [9], [10], [11] and space-time light sheets with arbitrary group velocities [12], [13], [14], [15]. These breakthroughs open new frontiers for applications involving space-time beams, including laser processing [16], complex light–matter interactions [17], and optical communications [18].

The Moiré effect, characterized by interference patterns resulting from the superposition of two similar but distinct periodic structures, was utilized for high-precision displacement and angular measurements due to the low-frequency fringes it produces [19]. Recently, this phenomenon has garnered significant attention across various physical systems due to its connection with extremely flat spectral bands, which are characterized by minimal energy dispersion and provide an ideal platform for exploring exotic quantum states [20], [21], [22], [23]. Although Moiré patterns are conventionally observed in real space by overlapping crystalline structures at different initial angles, their complexity simplifies when viewed in momentum space. For instance, a hexagonal Moiré lattice can be understood as the superposition of two spectra, each with six-fold discrete rotational symmetry and a specific angular offset. This concept enables the generation of spatially structured light fields that exhibit crystal and Moiré lattice, characterized by diffraction-free properties and high tunability [24], [25]. However, given the distinct characteristics of space-time beams compared to conventional spatially structured light, the potential to generate spatiotemporal Moiré lattice light fields through joint space-time modulation remains an intriguing question.

Here, we theoretically propose and experimentally demonstrate the generation of spatiotemporal Moiré lattice light fields. We show that these fields can theoretically be achieved by controlling the discrete rotational symmetry of the pulse’s spatiotemporal spectrum. Based on this principle, we experimentally generate and characterize spatiotemporal Moiré lattice beams with different sublattice sizes using a custom 4*f* pulse shaper [2] and our previously proposed *x* − *ω* space-time modulation strategy [7]. We also show the impact of the angular offset in the spatiotemporal spectra on the resulting spatiotemporal Moiré lattice light fields. Furthermore, we demonstrate that these light fields exhibit diffraction-free behaviour in their time-integrated intensities. Finally, we generate spatiotemporal Moiré lattice light fields that carry transverse orbital angular momentum, by introducing extra phase modulation into their spatiotemporal spectra. Our results provide a pathway for studying the Moiré effect in the space-time domain and could be extended to other wave-based physical systems, such as acoustic and electron wave.

## 2 Results

Similar to the spatial Moiré light fields [25], the spectra of spatiotemporal Moiré lattice light fields can be theoretically expressed as:

(1) $$\begin{array}{c} \tilde{E}\left(k_{x},\Omega \right)={\tilde{E}}_{1}\left(k_{x},\Omega ,{\tilde{\theta }}_{1}\right)+{\tilde{E}}_{2}\left(k_{x},\Omega ,{\tilde{\theta }}_{2}\right),\\ {\tilde{E}}_{1}\left(k_{x},\Omega ,{\tilde{\theta }}_{1}\right)=\overset{N-1}{\underset{n}{\sum }}\delta \left[k_{x}-R_{0}\cos\left(n\cdot \theta_{N}+{\tilde{\theta }}_{1}\right),\Omega -R_{0}\sin\left(n\cdot \theta_{N}+{\tilde{\theta }}_{1}\right)\right],\\ {\tilde{E}}_{2}\left(k_{x},\Omega ,{\tilde{\theta }}_{2}\right)=\overset{N-1}{\underset{n}{\sum }}\delta \left[k_{x}-R_{0}\cos\left(n\cdot \theta_{N}+{\tilde{\theta }}_{2}\right),\Omega -R_{0}\sin\left(n\cdot \theta_{N}+{\tilde{\theta }}_{2}\right)\right]. \end{array}$$

Here, *k*_*x*_ is the spatial frequency, $\Omega =\gamma \left(\omega -\omega_{0}\right)$ is the detuning temporal frequency, *ω* is the time frequency, *ω*_0_ is the central frequency, *γ* = Δ*k*_*x*_/Δ*ω* is the reduction coefficient ensuring consistency between the spatial and temporal scales, and *N* is an arbitrary positive integer corresponding to *N*-fold discrete rotational symmetry. Restricted by the light cone equation $k_{x}^{2}+k_{z}^{2}={\left(\omega /c\right)}^{2}$ , one can see that the longitudinal wave number (propagation constant) of space-time beams broadens with the temporal spectrum Δ*ω*, resulting in Δ*k*_*z*_. ${\tilde{E}}_{1}$ and ${\tilde{E}}_{2}$ are two sets of impulse spectral components, each exhibiting *N*-fold discrete rotational symmetry but with different initial angles ( ${\tilde{\theta }}_{1}$ and ${\tilde{\theta }}_{2}$ ). These components correspond to two original spatiotemporal lattice fields in the space-time domain. *δ* represents the Dirac delta function, *R*_0_ is related to the space-time spectral width, and *θ*_*N*_ = 2*π*/*N*. By adjusting the shift angle $\Delta \tilde{\theta }={\tilde{\theta }}_{1}-{\tilde{\theta }}_{2}$ between the two original spatiotemporal lattices, the sub-lattice size (periodicity) of the Moiré lattice can be controlled, as we will demonstrate below. It is important to note that Eq. (1) implicitly assumes *N* = 2, 3, 4, and 6 to ensure translational symmetry of the two original lattices forming the Moiré lattice, i.e., guaranteeing crystalline symmetry [26]. Conversely, for other values of *N*, a quasicrystalline pattern emerges. By applying an inverse spatiotemporal Fourier transform to Eq. (1), the spatiotemporal Moiré lattice light fields can be obtained, i.e., $E\left(x,z,t\right)=\iint \tilde{E}\left(k_{x},\Omega \right){\text{e}}^{\text{i}\left(kx-\Omega t\right)}\text{d}k_{x}\text{d}\Omega$ .

**Figure 1:**
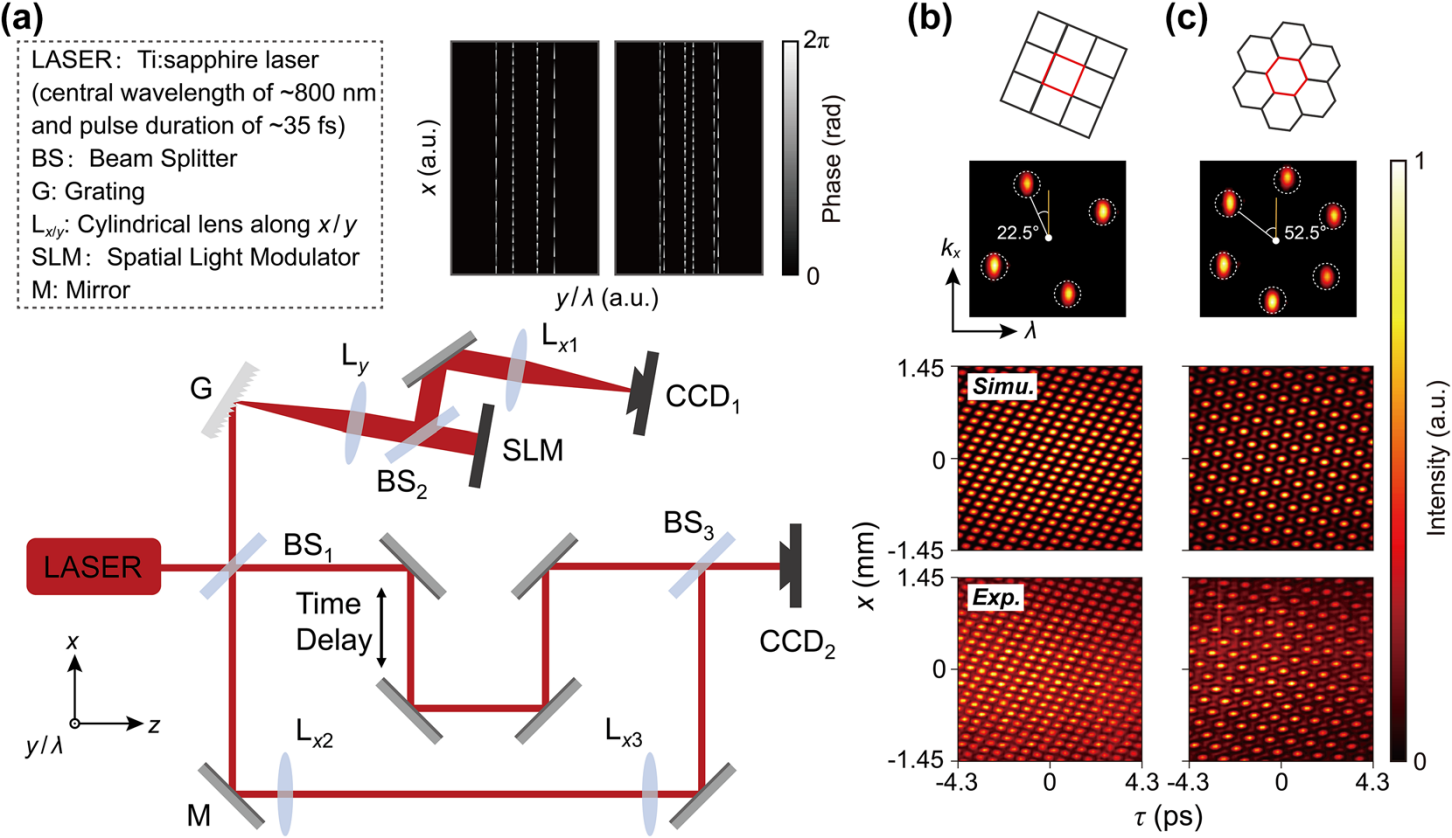
Experimental setup and generation of spatiotemporal crystal lattice light fields. (a) Experimental setup consists of three sections: (1) the space-time beam generator comprising of a grating, a cylindrical lens *L*_*y*_ and a spatial light modulator (SLM); (2) the spatiotemporal Fourier spectra analyser, which includes a cylindrical lens *L*_*x*_ and a CCD camera (CCD_1_); and (3) the time-resolved profile measurement section, realized via a Mach–Zehnder interferometer consisting of two BSs (BS_1_ and BS_3_), a 4*f* system including two cylindrical lenses *L*_*x*2_ (*f* = 400 mm) and *L*_*x*3_ (*f* = 200 mm), a CCD camera (CCD_2_) placed on the back focal plane of *L*_*x*3_, and a motorized translation stage in the reference path. Two SLM phase patterns for different *N*-fold discrete rotational symmetry modulation (*N* = 4, 6) are loaded. (b) The simplified graph on the top of the column reflects such structure more vividly, with black grid lines representing the structure of the spatiotemporal lattice and red lines highlighting a single lattice cell. The measured spectrum and intensity distribution for the space-time beam with four-fold discrete rotational symmetry (the initial angle is set to 22.5°) are shown, with good agreement between the experiment (Exp.) and simulation (Simu.). (c) Similar to (b) but for *N* = 6, with the initial angle set to 52.5°.

The experimental setup for generating spatiotemporal Moiré lattice light fields is illustrated in Figure 1(a). Femtosecond pulses from a Ti:sapphire laser, centred at a wavelength of ∼800 nm, are tailored using a custom 4*f* pulse shaper with a 2D phase-only spatial light modulator (SLM, PLUTO-2.1-NIR-133, Holoeye). The beam is then analysed using a Mach–Zehnder interferometer. The optical frequencies *ω* (or the wavelengths *λ*) of the reshaped beam are collimated along the horizontal *y*-axis using a diffraction grating (1,800 lines/mm, GH25-18V, Thorlabs) and a cylindrical lens (*L*_*y*_, *f* = 100 mm). The phase patterns loaded into the SLM (shown in Figure 1(a)) are calculated via Eq. (1), assigning a specific *ω* (or *λ*) to each spatial frequency $\left|k_{x}\right|$ in the Fourier plane of *L*_*y*_, according to our previously proposed *x* − *ω* space-time modulation strategy [7].

To measure the spatiotemporal spectra ${\left|\tilde{E}\left(k_{x},\Omega \right)\right|}^{2}$ , the light field in the pulse shaper is partially reflected by BS_2_ and directed through a spherical lens (*L*_*x*_, *f* = 100 mm) to perform a spatial Fourier transform along the *x*-axis onto a CCD (CCD_1_, SP620U, Ophir). To acquire the time-resolved light field ${\left|E\left(x,z;\tau \right)\right|}^{2}$ , a Mach–Zehnder interferometer is used, where the reference pulse is derived from the former pulses via BS_1_ and reshaped to ∼100 fs using a Gaussian spectral filter (Δ*λ* = 10 nm) to balance the trade-off between the contrast of interference fringes and reconstruction resolution (see the Supplementary Information Section 1 for details). The space-time beam, which is transported by a 4*f* system including cylindrical lenses *L*_*x*2_ (*f* = 400 mm) and *L*_*x*3_ (*f* = 200 mm), overlaps with the reference pulse, generating interference fringes that are captured by another CCD (CCD_2_) placed on the back focal plane of *L*_*x*3_. Since the space-time beam is temporally longer, its temporal slice can be reconstructed from the fringes at a specified time delay *τ*. The generated spatiotemporal light fields can be reconstructed by using the reference pulse to scan in the time domain and collecting all corresponding temporal slices.

We first generate two spatiotemporal crystal lattice light fields with four-fold (*N* = 4) and six-fold (*N* = 6) discrete rotational symmetry. The measured spatiotemporal spectra ${\left|\tilde{E}\left(k_{x},\Omega \right)\right|}^{2}$ for these two lattice light fields are shown in the second row of Figure 1(b) and (c). Without loss of generality, the initial angles in their spatiotemporal spectra relative to the Ω-axis are set to 22.5° and 52.5°, respectively. It can also be observed that the spatial and temporal bandwidths are Δ*k*_*x*_ = ∼66 rad/mm and Δ*λ* = ∼5 nm, respectively. Figure 1(b) and (c) also present the simulated (the third row) and measured (bottom row) time-resolved light fields ${\left|E\left(x,z;\tau \right)\right|}^{2}$ . There is good agreement between theory and experiment, clearly showing that *N* = 4 and *N* = 6 correspond to square and hexagonal lattice patterns, respectively. Moreover, these patterns exhibit rotation angles that are positively correlated with the initial angle of their spatiotemporal spectra (see Supplementary Information Section 2 for details). The slight variation in rotation angles between the space-time and spectral domains is due to the different scaling of the coordinate axes.

**Figure 2:**
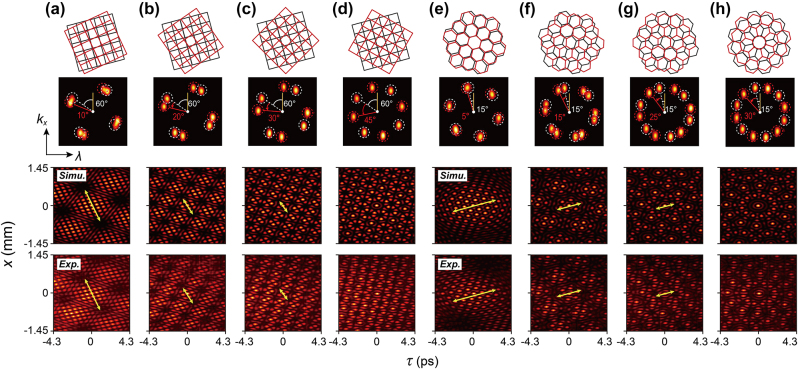
Generation and characterization of spatiotemporal Moiré lattice light fields. (a)–(d) Spatiotemporal Moiré lattice light fields by superimposing two spatiotemporal lattice fields with four-fold discrete rotational symmetry (*N* = 4). The top row illustrates the simplified lattice structures, with black grid lines representing the structure of one lattice and red lines for the other. The second row shows the measured spatiotemporal spectra, where the initial angle of one spectrum (white dashed circles) is fixed at 60°, and the shift angles of the second spectrum (red dashed circles) are set to 10°, 20°, 30°, and 45°. The third and fourth rows display the calculated and measured time-resolved light field distributions, respectively, where the yellow double arrows denote the sublattice orientation and size. (e)–(h) Similar to (a)–(d) but for *N* = 6, with the initial angle set to 15° and the shift angles are 5°, 15°, 25° and 30°, respectively.

Next, we generate spatiotemporal Moiré lattice light fields as described by Eq. (1). Figure 2(a)–(d) present the simulated and measured results for Moiré lattice light fields via superimposing two sets of spatiotemporal crystal lattices with four-fold discrete rotational symmetry. Notably, we fix the initial angle of one set’s spatiotemporal spectra ${\left|{\tilde{E}}_{1}\left(k_{x},\Omega ,{\tilde{\theta }}_{1}\right)\right|}^{2}$ (indicated by white dashed circles) relative to the Ω-axis at 60°, while shifting the other set ${\left|{\tilde{E}}_{2}\left(k_{x},\Omega ,{\tilde{\theta }}_{2}\right)\right|}^{2}$ (indicated by red dashed circles) by $\Delta \tilde{\theta }$ values of 10°, 20°, 30°, and 45°, respectively. It can be observed that $\Delta \tilde{\theta }$ provides an effective degree of freedom for controlling the spatiotemporal Moiré lattices. Specifically, the generated spatiotemporal Moiré lattices exhibit the typical angle-size relationship: as the rotation angle increases, the size of the sublattice (highlighted by yellow double arrows) decreases [20], [21]. This phenomenon offers a vivid perspective on visualizing the phase transition from a crystal (*N* = 4) to quasicrystal (*N* = 8). A similar phenomenon is observed in the spatiotemporal Moiré lattices generated by superimposing two sets of spatiotemporal crystal lattice light fields with six-fold discrete rotational symmetry. As the shift angle $\Delta \tilde{\theta }$ increases through 5°, 15°, 25°, and 30°, the complexity of the resulting spatiotemporal patterns rises rapidly, reflecting the phase transition from a hexagonal lattice (*N* = 6) to a quasicrystal (*N* = 12). Furthermore, all experimental results show good agreement with the theoretical predictions.

**Figure 3:**
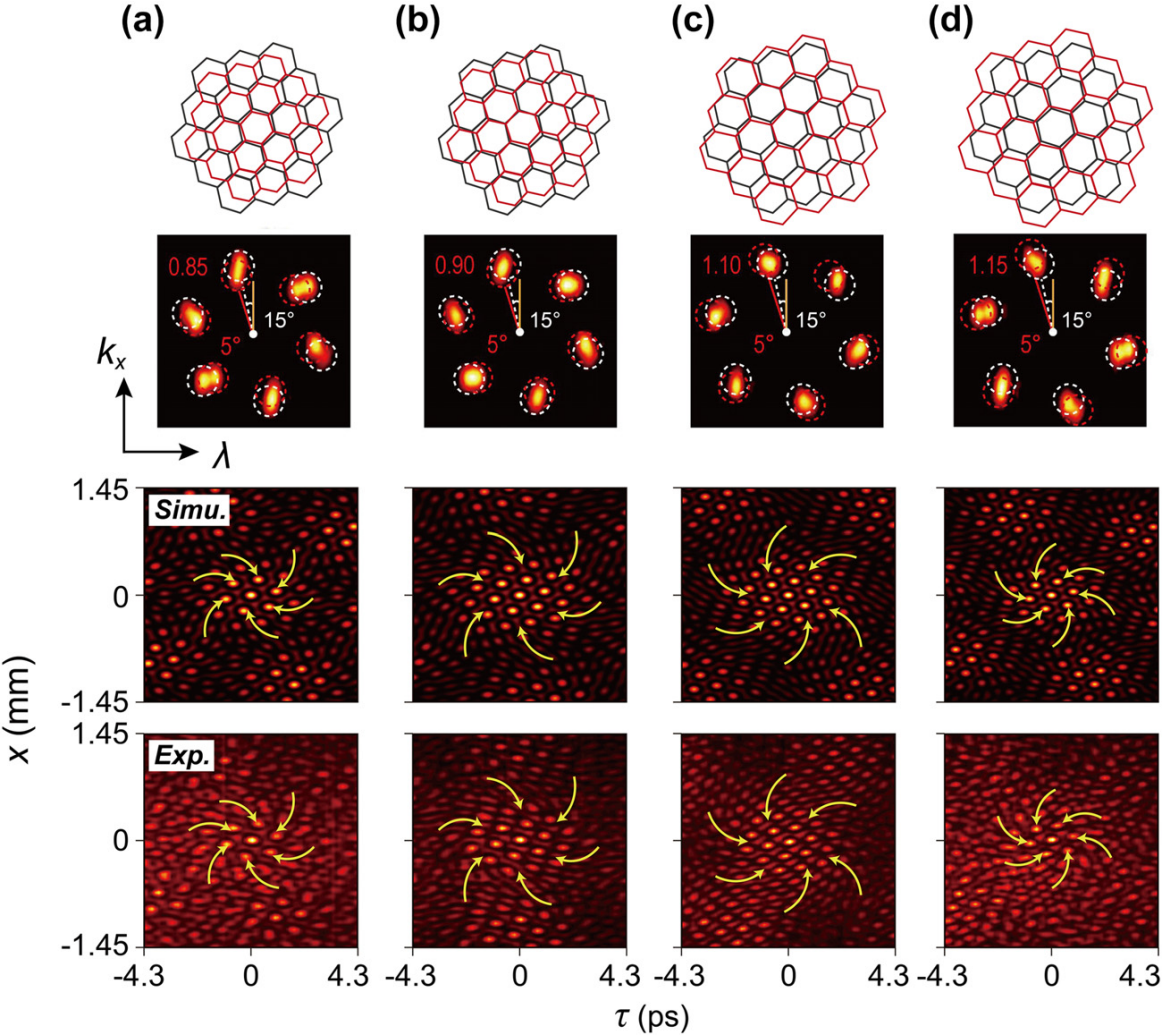
Generation and characterization of spatiotemporal Moiré-like lattices with varying spectral width ratios. (a)–(d) The simplified diagrams at the top illustrate the Moiré-like structures generated by superimposing two spatiotemporal crystal lattice light fields with six-fold discrete rotational symmetry, but differing lattice constants. The second row shows the measured spatiotemporal spectra, where the initial angle is set to 15°, and the rotation angle between the two lattices is fixed at 5°. The spectral width ratios of the second crystal lattice to the first are *r* = 0.85, 0.90, 1.10, and 1.15, respectively, and two sets of spectra are indicated by the red and black dashed circles. The third and fourth rows present the calculated and measured intensity distributions of the spatiotemporal Moiré-like lattices, where the yellow curve arrows represent the twist directions.

We also synthesize spatiotemporal Moiré-like lattices by superimposing two spatiotemporal crystal lattice light fields, both with six-fold discrete rotational symmetry (*N* = 6) but varying lattice constants (or different spectral widths), while maintaining a constant shift angle of $\Delta \tilde{\theta }=$ 5°. In this case, we explore how varying the ratio between the spectral widths of the two crystal fields affects the resulting Moiré-like pattern. As shown in Figure 3(a)–(d), different spectral width ratios (*r* = 0.85, 0.9, 1.1, and 1.15) lead to distinct sublattice structures. When the spectral widths of the two superimposed fields are nearly equal (*r* ≈ 1), the resulting sublattice pattern is symmetric, with little twisting. As the ratio deviates further from 1, the sublattice size decreases, and the twisting becomes more noticeable. For *r* < 1, sublattices twist clockwise, while for *r* > 1, they twist anticlockwise. This variation in twist direction, marked by yellow curves in Figure 3(a)–(d), is directly influenced by the ratio of the spectral widths. This ability to fine-tune the sublattice size and orientation by adjusting the spectral properties of the individual spatiotemporal crystal fields open new possibilities for engineering complex, highly tunable light field patterns.

**Figure 4:**
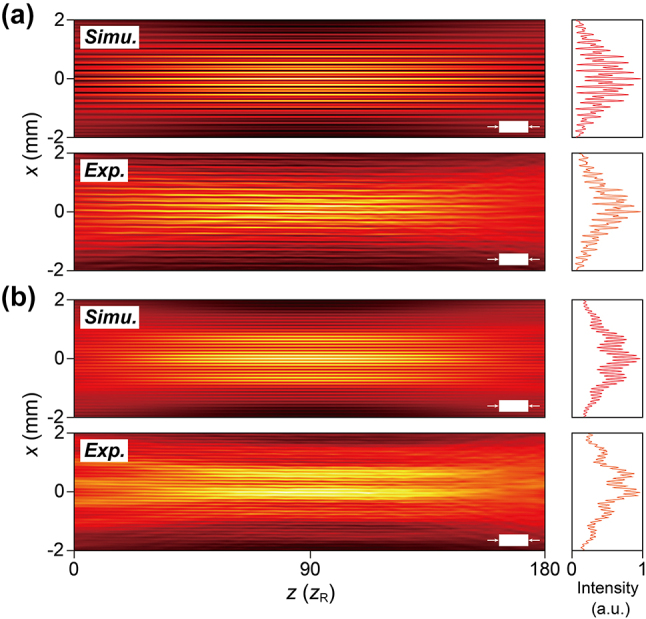
Diffraction-free propagation of spatiotemporal Moiré lattice light fields. (a) Simulated and experimental propagation of spatiotemporal Moiré lattices with four-fold discrete rotational symmetry (*N* = 4) and a shift angle of 30°. The propagation distance is up to ∼180 times the Rayleigh distance (*z*_R_) of a Gaussian beam with a full width at half maximum (FWHM) of ∼33 μm. The solid white blocks in the bottom-right corners represent 50 mm. On the right, the cross-sectional intensity profiles along the *x*-axis at *z* = 90*z*_R_ are shown. (b) Similar to (a) but for *N* = 6 with the shift angle of 15°.

Notably, the time-averaged profile of spatiotemporal Moiré lattice light fields, ${\left|E\left(x,z\right)\right|}^{2}=\int {\left|E\left(x,z;\tau \right)\right|}^{2}\text{d}\tau$ , exhibits diffraction-free propagation characteristics. Generally, the time-averaged profile of a spatiotemporal beam can be expressed as [15]:

(2) $$\begin{array}{ll} {\left|E\left(x,z\right)\right|}^{2} & =\int {\left|E\left(x,z;\tau \right)\right|}^{2}\text{d}\tau \\ & =\iint H\left(k_{x},k_{x}^{\prime };z\right){\text{e}}^{\text{i}\left(k_{x}-k_{x}^{\prime }\right)x}\text{d}k_{x}\text{d}k_{x}^{\prime }, \end{array}$$

where $H\left(k_{x},k_{x}^{\prime };z\right)=\int \tilde{E}\left(k_{x},\Omega \right){\tilde{E}}^{*}\left(k_{x}^{\prime },\Omega \right){\text{e}}^{\text{i}\left(k_{z}-k_{z}^{\prime }\right)z}\text{d}\Omega$ , $k_{z}=\sqrt{{\left(\omega /c\right)}^{2}-k_{x}^{2}}$ , and $k_{z}^{\prime }=\sqrt{{\left(\omega /c\right)}^{2}-k_{x}^{\prime 2}}$ . Here, *k*_*x*_ and $k_{x}^{\prime }$ represent any two spectral components in the spatiotemporal spectrum. It is easy to see that if the spatiotemporal spectrum takes the form $\tilde{E}\left(k_{x},\Omega \right)=\tilde{E}\left(k_{x}\right)\delta \left(\Omega -\Omega \left(\left|k_{x}\right|\right)\right)$ , then $H\left(k_{x},k_{x}^{\prime };z\right)=H\left(k_{x},k_{x}^{\prime };0\right)$ , leading to ${\left|E\left(x,z\right)\right|}^{2}={\left|E\left(x,0\right)\right|}^{2}$ . Clearly, spatiotemporal Moiré lattice light fields satisfy this condition because their spatiotemporal spectrum Ω is locked to a specific spatial frequency $\left|k_{x}\right|$ , as described by Eq. (1). Consequently, their time-averaged intensities can propagate without significant diffraction. Additionally, the evolution of the ST lattice fields during propagation can be explained using the light cone theory [12] (see the Supplementary Information Section 3 for details), but this evolution does not affect their time-integrated intensity propagation invariance. We thereby carry out experiments to confirm the diffraction-free properties of spatiotemporal Moiré lattice light fields. Figure 4(a) and (b) present a comparison between the simulated and experimental results for the generated spatiotemporal Moiré lattices. These profiles were measured over a propagation distance of up to ∼180 times the Rayleigh distance *z*_R_ of a Gaussian beam with a full width at half maximum (FWHM) of ∼33 μm. The corresponding intensities at *z* = 90*z*_R_ on the right-hand side of each plot further highlight the consistency between the experimental (red) and simulated (orange) results, showing that the intensity distribution remains stable over the entire propagation range.

**Figure 5:**
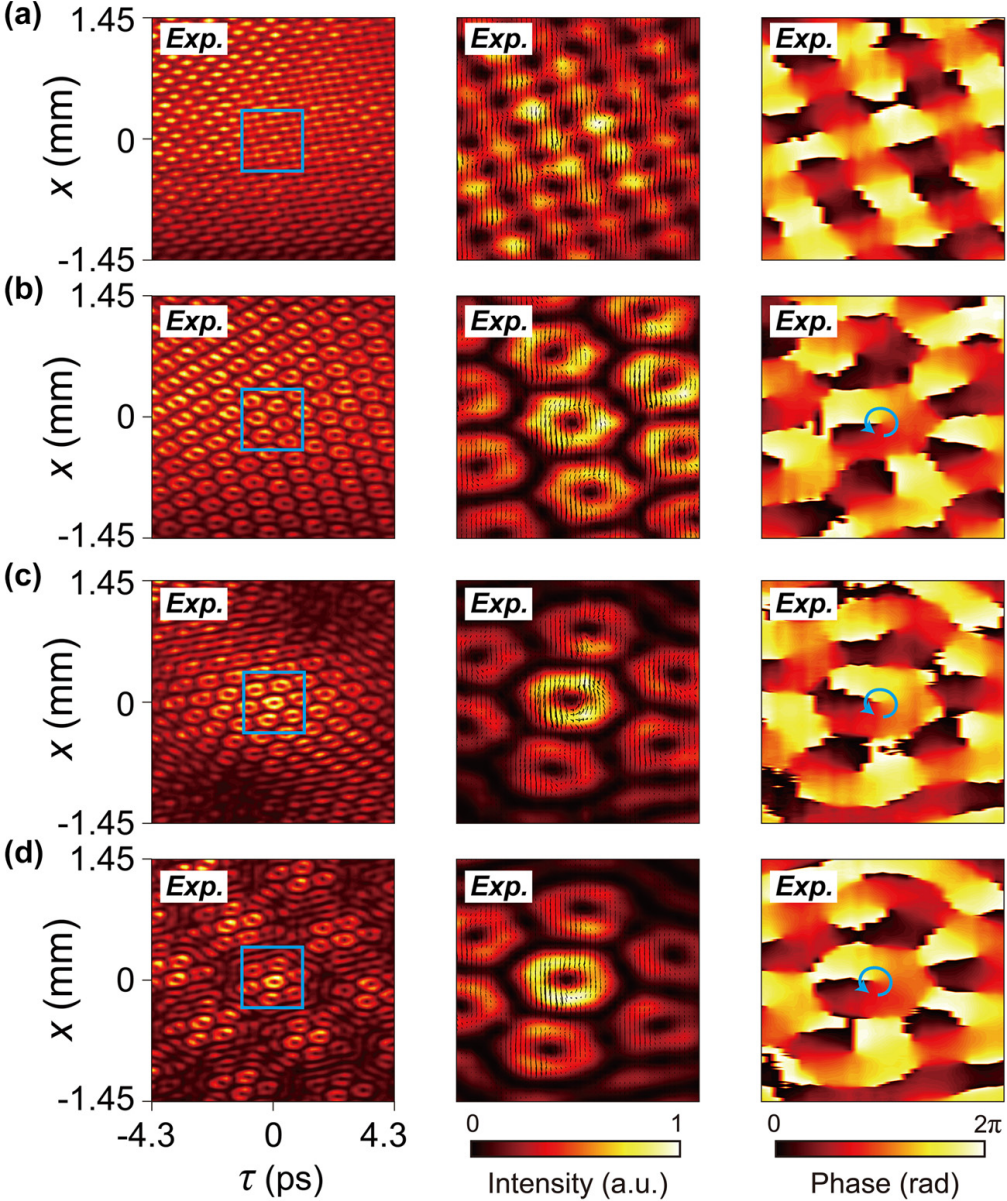
Phase modulation in spatiotemporal crystal and Moiré lattice light fields. (a)–(d) The four rows correspond to *N* = 4 and 6 spatiotemporal crystals, and spatiotemporal Moiré lattices with shift angles of 5° and 15°, respectively. The left column shows the experimentally measured intensity distributions of the space-time beams. The regions inside the blue boxes are magnified and displayed in the second column, showing the measured intensity distributions with calculated Poynting vectors indicated by the black arrows. The right column presents the measured phase distributions, where the blue arrows indicate the direction of phase increase, confirming the formation of spatiotemporal vortex arrays with a topological charge of *ℓ* = 1.

So far, the results we have presented are based on spatiotemporal spectra where all frequency components share the same phase. By modulating the momentum space (or spatiotemporal spectra), we can obtain more complex spatiotemporal lattice light fields by adjusting the relative phases of different frequency components. A typical example is introducing a spiral phase with a topological charge *ℓ* = 1, e^i*φ*^ (where $\varphi =\text{atan}\left(ck_{x}/\Omega \right)$ is the azimuthal angle in the spatiotemporal spectral domain, and *c* is the light speed in vacuum), to generate a spatiotemporal vortex lattice that carries transverse orbital angular momentum. We first applied the spiral phase e^i*φ*^ to a spatiotemporal square lattice field with *N* = 4. However, due to sparse frequency sampling, the vortex structure did not form effectively, resulting in a checkerboard-like intensity distribution (left plot of Figure 5(a)). Simulations reveal that the corresponding phase is a localized *π*-step across the grid (right plot of Figure 5(a)). As the spectral complexity increases (i.e., with a larger *N*), localized spatiotemporal vortexes with their respective intensity singularities emerges, as shown in the case of *N* = 6 (Figure 5(b)). This vortex structure can further be tailored using Moiré engineering by adjusting the rotation angle of the Moiré patterns (Figure 5(c) and (d), corresponding to original Moiré light fields in Figure 2(e) and (f)). The simulated spatiotemporal intensity and phase distributions (the right plots of in Figure 5(b)–(d)) and the calculated Poynting vectors ( $i\left(E\nabla E^{*}-E^{*}\nabla E\right)/2$ , where *E* is the amplitude of the space-time beams [27]) confirm the formation of a spatiotemporal vortex array with a topological charge of *ℓ* = 1.

## 3 Conclusions

In conclusion, we theoretically describe and experimentally generate spatiotemporal Moiré lattice light fields. We demonstrate that these fields are constructed by superimposing two sets of spatiotemporal spectra with discrete rotational symmetry and a specific shift angle. Our results show that the shift angle is inversely proportional to the size of the sublattices in the spatiotemporal Moiré lattice light fields. Additionally, we demonstrate that the ratio of the spectral widths of the two original spatiotemporal spectra provides an extra degree of freedom for tailoring the distribution of these Moiré lattices. Furthermore, by modulating the phase of the spatiotemporal spectra, we introduce even greater complexity, such as generating spatiotemporal vortex arrays that carry transverse orbital angular momentum.

Future research could explore the interaction between these space-time beams and artificial microstructures, such as nonlinear optical superlattices [28], [29] and liquid crystals [30], [31]. By combining the control of symmetry in spatiotemporal spectra with complex amplitude modulation strategies, a broader range of novel spatiotemporal beams may be uncovered [32], [33]. While our experimental setup is effective and easy to operate, more compact or even on-chip schemes are potentially developed, particularly using metasurfaces that simultaneously control spatial and temporal frequencies [34], [35], [36]. To date, the regulation of frequency symmetry to generate Moiré structures is not yet widely utilized for space-time beam manipulation, and our work highlights the versatility of this method as an efficient platform for controlling the spatiotemporal dynamics of pulsed light fields. Finally, we believe this scheme applies to other wave systems, such as acoustic [37], [38], water waves [39], and even electron waves [40], opening new avenues for the study and application of these beams across a wide range of fields.

## Supplementary Material

Supplementary Material Details
