# Supplementary material for: Spatiotemporal Moiré lattice light fields
Source: Nanophotonics. 2025 Jan 6;14(6):707–14. doi: 10.1515/nanoph-2024-0562 (PMC11964129; doi:10.1515/nanoph-2024-0562)
Supplement: Supplementary file 1 — Supplementary Material Details [file j_nanoph-2024-0562_suppl_001.pdf]

Supplementary Information

Spatiotemporal Moiré lattice light fields

An-Zhuo Yu[1,#], *Wang Zhang*[1,#], *Wei Chen*[1,*], *Yuan Liu*[1,*], *Chao-Qun Ma*[1], *Jia-Chen Yang*[1], *Yan-Qing Lu*[1,*]

[1] National Laboratory of Solid State Microstructures, Key Laboratory of Intelligent Optical Sensing and Manipulation, Collaborative Innovation Center of Advanced Microstructures, College of Engineering and Applied Sciences, Nanjing University, Nanjing 210023, China,
Corresponding to:
*E-mail: wchen@nju.edu.cn
*E-mail: liuyuan@nju.edu.cn
*E-mail: yqlu@nju.edu.cn
#These authors contributed equally to this work.

The Supporting Information includes:

1. Pulse duration selection
2. Impact of the angular offset on results
3. Propagation dynamics via the light cone theory

1. Pulse duration selection

The pulse width of the laser source is 35 fs, with its corresponding spectral bandwidth (FWHM) of **~40 nm**, while the spectral bandwidth of our ST lattice light field is set to **~5 nm**. This **mismatch** in spectral bandwidths between signal and reference pulses causes a majority of reference pulse spectral components unrelated to interference to contribute a bright background, thereby lowering the contrast of interference fringes and complicating the ST beam reconstruction process. On the other hand, the reference pulse duration has to be not too large, so that improves the reconstruction resolution and endows the reconstruction with more fine details. Considering this trade-off, the pulse of the laser source is reshaped to ~100 fs (by a spectral filter with bandwidth of ~10 nm), ensuring sufficient contrast of interference fringes and a relatively narrow pulse width at the same time.

2. Impact of the angular offset on results

The discrete points in the spatiotemporal spectrum are associated with distinct spatial and temporal frequencies due to varying angular offsets, which in turn affect the periods along the x- and τ- axes, respectively. More specifically, changing the angular offset results in the simultaneous rotation of both the spatiotemporal spectrum and the ST lattice field at the same angle. To further demonstrate this point, we simulate the ST lattice of $N = 4$ as an example (Figure S1). The angular offset is initially set to 22.5°, and the ST lattice field is rotated by the same angle. When the offset is increased to 45°, both the spatiotemporal spectrum and the ST lattice field rotate accordingly by the same amount.

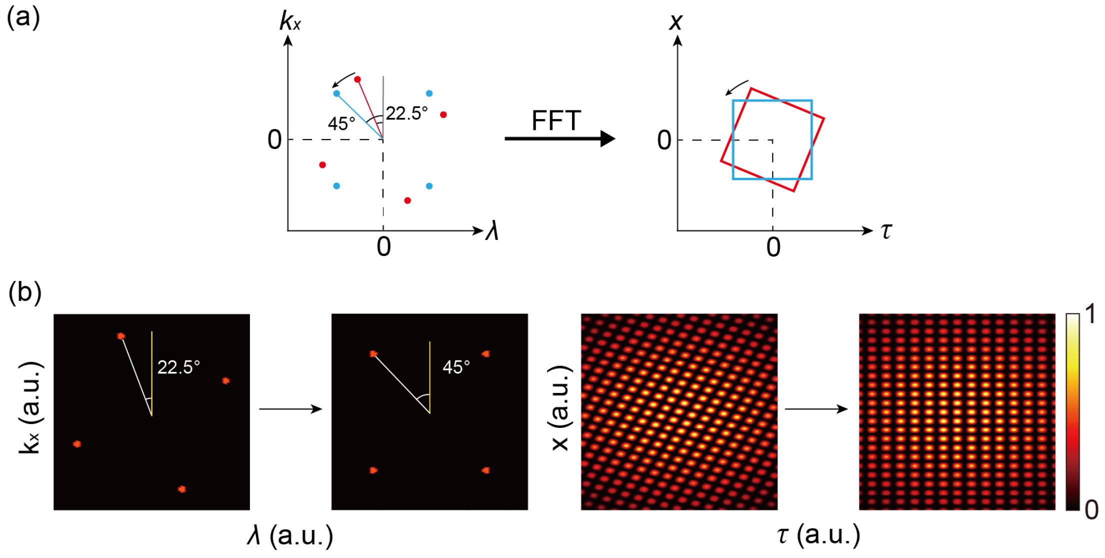

Figure S1. (a) The simplified graph of the spatiotemporal spectrum and the ST lattice field, with red points and lines representing the spectrum and a single lattice cell, respectively, of the ST lattice of $N = 4$ at an angular offset of 22.5°, while the blue for an angular offset of 45°. (b) Simulated spatiotemporal spectrums and the ST lattice fields for angular offsets of 22.5° and 45°, respectively.

3. Propagation dynamics via the light cone theory

The evolution of the ST lattice fields during propagation could be well described by the light cone theory [1].

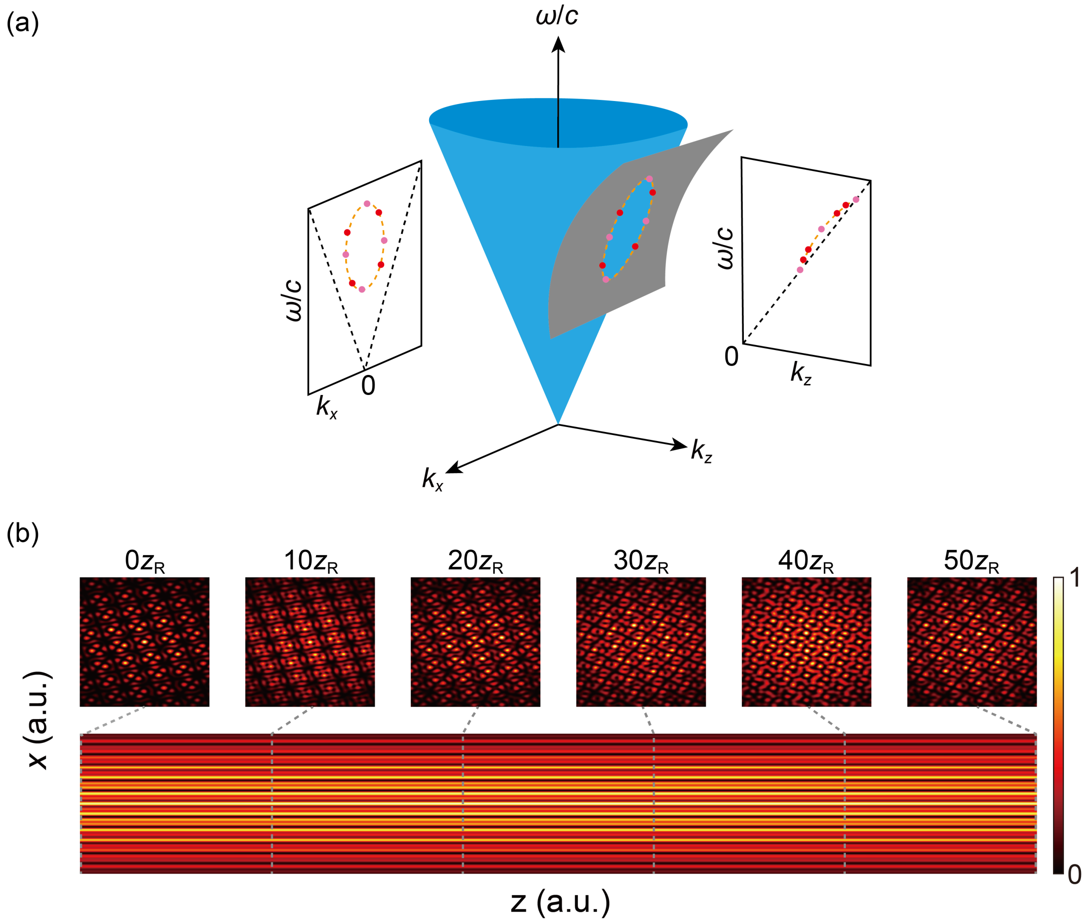

Figure S2. (a) Schematic of the light cone of the ST Moiré lattice light field of $N = 4$ and a shift angle of 30°. The spectrum lies on the trajectory (labelled by orange dashed circles) of the light cone and a curved plane representing the pulse coupling to a normal-GVD. The red circles are one set of ST lattices and pink circles for the other. The $\omega/c - k_x$ and $\omega/c - k_z$ projections are plotted on the left and right side, respectively. (b) The evolution of the ST lattice of a propagation distance of $0z_R$, $10z_R$, $20z_R$, $30z_R$, $40z_R$ and $50z_R$, labelled by gray dashed lines in the calculated time intensity distribution during the propagation, respectively.

We first consider the simplest case, that a 2D propagation-invariant monochromatic beam's spectrum must conform to a circularly symmetric distribution, such as Bessel beams [2]. In this case, the transverse wavenumber k_T is a constant, resulting in the longitudinal wavenumber $k_z = \sqrt{(\omega_0/c)^2 - {k_T}^2}$ = const, where ω_0 is the frequency and c is the light speed in vacuum. The constancy of k_z guarantees rigid propagation.

However, the situation is fundamentally different for the ST lattice light field with various spectral components ω. Here, k_z becomes frequency-dependent, following $k_z(\omega) =$

$\sqrt{(\omega/c)^2 - {k_x}^2}$, with $k_y = 0$. Changes in k_z values can also be elucidated clearly via the model of the light cone (Figure S2(a)). To form a circle-shaped trajectory consistent with the ST lattice spectra, the plane is curved, representing that the pulse is coupled to a normal-GVD (group-velocity dispersion) [3, 4]. And the $\omega/c - k_z$ projection further confirms that k_z ranges along the ω/c axis, leading to observable temporal evolution during ST lattice propagation. More specifically, it means that the free-space propagation of ST lattices corresponding to intersections of the light cone and such curved planes, would imitate propagation in a normal dispersive medium. As an example, Figure S2(b) presents the calculated evolution of the ST Moiré lattice light field of $N = 4$ and a shift angle of 30°, over a propagation distances of $0z_{\mathrm{R}}$, $10z_{\mathrm{R}}$, $20z_{\mathrm{R}}$, $30z_{\mathrm{R}}$, $40z_{\mathrm{R}}$ and $50z_{\mathrm{R}}$, respectively. It should be noted that the temporal evolution does not conflict with the time-integrated intensity propagation-invariant property, as we demonstrated in the manuscript (see also Figure S2(b)).

Reference:

[1] H. E. Kondakci and A. F. Abouraddy, "Diffraction-free space–time light sheets," *Nature Photonics,* vol. 11, no. 11, pp. 733-740, 2017/11/01 2017, doi: 10.1038/s41566-017-0028-9.

[2] J. a. M. Durnin, J. J. and Eberly, J. H, "Diffraction-free beams," *Physical Review Letters,* vol. 58, no. 15, pp. 1499-1501, 1987, doi: 10.1103/PhysRevLett.58.1499.

[3] M. Yessenov, L. A. Hall, K. L. Schepler, and A. F. Abouraddy, "Space-time wave packets," *Adv. Opt. Photon.,* vol. 14, no. 3, pp. 455-570, 2022/09/30 2022, doi: 10.1364/AOP.450016.

[4] L. A. Hall and A. F. Abouraddy, "Canceling and Inverting Normal and Anomalous Group-Velocity Dispersion Using Space-Time Wave Packets," *Laser & Photonics Reviews,* vol. 17, no. 3, p. 2200119, 2023, doi: https://doi.org/10.1002/lpor.202200119.
